# Preliminary analysis of single-nucleotide polymorphisms in IL-10, IL-4, and IL-4Rα genes and profile of circulating cytokines in patients with gastric Cancer

**DOI:** 10.1186/s12876-018-0913-9

**Published:** 2018-12-10

**Authors:** Denny Miley Cárdenas, Angie Carolina Sánchez, Daris Angélica Rosas, Esmeralda Rivero, Massiel Dayana Paparoni, Mildred Andreína Cruz, Yeicy Paola Suárez, Nestor Fabián Galvis

**Affiliations:** Faculty of Health Sciences, BIOGEN Research Group, Universidad de Santander, Avenida 4 10N-61, El Bosque, Cúcuta, Colombia

**Keywords:** Genetic polymorphism, Cytokines, Neoplasia

## Abstract

**Background:**

Gastric Cancer is highly prevalent and deadly worldwide. In Colombia, it is the most lethal form of cancer. Some single-nucleotide polymorphisms in IL-10, IL-4, and IL-4Rα genes have been associated with an anti-inflammatory environment and a Th2 profile in detriment of the antitumor Th1 response. This research sought to detect single-nucleotide polymorphisms in promoter sequences, like − 1082 (G/A), − 592 (C/A), and − 819 (C/T), as well as − 590 (C/T) of the IL-10 and IL-4 genes, respectively; in addition to the IL-4Rα mutation variants, Ile50Val and Q576R, together with circulating levels of IL-4, TNF-α, IL-10, and IFN-γ in patients with gastric carcinoma in Cúcuta, Colombia.

**Methods:**

In a cross-sectional study, 17 patients and 30 healthy individuals were genotyped for the six polymorphisms mentioned through PCR-RFLP of DNA obtained from peripheral blood cells and serum samples were analyzed by sandwich ELISA to quantify cytokines. Statistical difference between groups was determined along with the association between the presence of polymorphisms and the risk of gastric cancer, as well as the mortality in patients, using Mann-Whitney U test and logistic regression analysis, respectively.

**Results:**

An association between the − 1082 (G/A) and the risk of gastric cancer was found (OR = 7.58, range 0.77–74.06, *P* = 0.08). Furthermore, patients had a significant increase in IL-4 serum levels (*P* < 0.01) compared to healthy individuals, both variables showed a higher estimated risk of mortality in patients, although without statistical association (*P* > 0.05).

**Conclusion:**

We infer that two possible biomarkers (one immunological and one genetic) could be considered in association with gastric cancer in our population, which should be confirmed by subsequent studies involving a greater number of individuals.

## Background

Neoplasms represent serious morbidity and mortality problems globally, especially in developing countries, which account for 57% of the cases and 65% of the associated deaths, with gastric cancer among the five most-incidental and lethal; this is twice as frequent in men as in women [1]. In Colombia, gastric carcinoma is among the five most frequent neoplasms, prioritized by the 2012–2015 ten-year plan for Cancer Control, occupying the second and fourth place in men and women, respectively (although third in female population from the Colombian eastern region) [2, 3], but being the first in mortality, attributed with 17.6% of deaths, surpassing prostate (15.0%), lung (14.8%), and colorectal (6.5%) cancer in males, besides causing 11.5% of cancer-related deaths in female population (only preceded by breast and cervical cancer with close frequencies of 12.1 and 12.3%, respectively) [4]. This morbidity and mortality behavior for gastric cancer in Colombia is similar to that observed in other countries in Central and South America, like Costa Rica, Brazil, Chile, and Argentina [5], consolidated as a highly lethal neoplasia; a situation that, nevertheless, results modifiable through its early detection or during its initial stage [6].

The Colombian department of Norte de Santander is among the top 10 in mortality rate, with 163/4364 (3.74%) cases, especially in population >  50 years of age (according to the Statistical Yearbook by the National Cancer Institute, 2010), given that it is within the Andean region, a geographic zone concentrated by the Cancer Population Registry (RPC, for the term in Spanish) in Colombia [2].

Several point variations, like single-nucleotide polymorphism (SNP) in gene promoter sequences present in the human population, have been evaluated and correlated to the risk of developing neoplasms, such as lymphomas [7–11] or other solid tumors, like cervical, hepatocellular, and gastric carcinoma [12–14] with controversial results regarding the association with the risk of developing a given neoplasm, for example, have a better disease-free survival in patients who lack SNPs with altered IL-10 production, such as − 1082 (G/A) [15].

On the other hand, in patients with benign brain tumor it has been referred as a differential characteristic to healthy controls and those with malignant-type tumor, a higher prevalence of this latter SNP, which the authors suggest would be reflected in decreased IL-10 levels in plasma, confers a good prognosis [16].

The **−** 592 (C/A) SNP has been linked to overexpression of IL-10 and associated with the risk of developing Non-Hodgkin Lymphoma of B cells (NHL-B) [8] even in other pathologies, including gastric cancer [13].

An important role of Th2 profile cytokines, such as IL-4, in the polarization and activation of M2 macrophages (with tolerogenic function or incapable of activating the T lymphocyte response) has been described, favoring their pro-tumor function or neoplasm progression due to the production of IL-10 (among other molecules) that suppresses the function of other immune cells of great relevance as the cooperating (CD4+) and cytotoxic (CD8+) T lymphocytes [17, 18].

These infiltrating M2 macrophages in tumors, known as TAMs can be reduced by IFN-γ effect (enhanced by adding IL-12), as shown in vitro assays [19, 20], and which together with TNF-α, considered the most ubiquitous produced by the majority of subpopulations of memory T lymphocytes [21], are essential in the effector and protective immune response of the individual [22, 23]. In this regard, an association has been established between a SNP in (C to T) -590 in the promoter region of the IL-4 [24], leading to overexpression of this cytokine, with increased risk of developing cardiac subtype (OR = 2.44) or diffuse gastric carcinoma (OR = 1.64) [15]; this may be poorly differentiated and considered metastatic [25], in contrast with that reported, for example, for another point mutation variant in this same region, like − 168 (T/C), associated to lower risk of developing this neoplasia (cardiac subtype) [26].

The usefulness of detecting SNPs in promoters of immunoregulatory cytokine genes (IL-4 and IL-10) is unknown, as is its association with the risk of gastric cancer in our environment, which even later contribute to the prognostic classification, taking into account the level of these circulating cytokines detected in plasma, given reports of increased IL-4 levels [15] or IL-10 and decreased IFN-γ and IL-12 in association with poor prognosis in patients with lung, colorectal, hepatocellular, pancreatic, renal carcinoma, malignant melanoma, NHL-B and gastric carcinoma [27, 28], with high levels of IL-10 particularly for the latter [29–31].

This study sought to analyze the presence of point variants in the promoter region of both IL-10 and IL-4 genes, like − 1082 (G/A), − 819 (C/T), − 592 (C/A), and − 590 (C/T), respectively, and in the IL-4Rα sequence (Ile50Val and Q576R), simultaneously with circulating levels of IL-4, TNF-α, IL-10, and IFN-γ. The aforementioned under the hypothesis that the GCC haplotype could represent a possible increased production of IL-10 in patients with gastric cancer in our country, as reported by Mitsayasu in another population [32], and mutations reported for IL-4 and its receptor alpha chain either have increased production of this cytokine or a greater effect after its signaling, which could avoid the antitumor effector T response.

The aforementioned takes into account that this molecule promotes the deflection of the T response towards a Th2 profile (antagonic function with IL-12 and IFN-γ) with respect to Th1 and apparently exerts a positive effect on the induction of other cytokines, such as IL-10 [17, 18], so their analysis is of great interest in our study.

## Methods

Descriptive and cross-sectional study with a correlational level.

### Study population

The population selected consisted of two groups of individuals: patients with gastric cancer (newly diagnosed and preferably still without treatment) from health entities, such as: the Maternal-Infant Center for Specialized Diagnosis (CEDMI, for the term in Spanish), E.S.E. Erasmo Meoz University Hospital (HUEM, for the term in Spanish), Gastroquirurgica Ltda, and Northern Santander League Against Cancer, which voluntarily agreed to participate, along with a group of healthy individuals (controls) residing in the city of Cúcuta; the groups were matched by age and gender.

The sample was comprised by 17 patients with gastric carcinoma and 30 healthy individuals from the city of Cúcuta. They were enrolled during the period from November 2014 to June 2015 through non-probabilistic sampling (intentional way). The selection was based on the following criteria: adults cared for in a health institution linked to the study during the period of time mentioned, with confirmed diagnosis of gastric cancer (anatomopathological opinion); a recent diagnosis, preferably without current treatment (Patient Group). For the control group: adult individuals who were blood donor candidates or members of the community, without diagnosis of current disease or personal or family background of gastric carcinoma. In general, voluntary participation was guaranteed. Signed informed consent was obtained from all patients and healthy individuals to be included in the study.

All non-adult individuals, those with any other underlying neoplasia prior to gastric carcinoma or concomitant with it, as well as those with primary or secondary immunosuppression or who did not agree to participate (themselves or their relatives) were excluded from the study. The Sample size was subject to compliance with the criteria exposed, obtaining an approximate ratio of 1:2, patients: healthy individuals, in favour of increasing the study’s statistical power.

All participants were seropositive for *H. pylori* (Enzyme-Linked ImmunoSorbent Assay, ELISA, screening as part of a previous study; unpublished results). Present investigation had a comparable population.

### Sampling

Samples were collected using the phlebotomy technique during a given period of time between November 2014 and June 2015. Two tubes were used, the first with anticoagulant EDTA to obtain whole blood for DNA extraction and genomic analysis; a second sample was taken in dry tube to determine the cytokine profile in serum.

### Sample processing

After collecting the blood samples, these were transported to the laboratory at Universidad de Santander (UDES). Each red-capped tube was centrifuged at 3500 rpm for 10 min to obtain serum used to determine four cytokines. Each lilac-capped tube was centrifuged at 1500 rpm for 20 min to obtain buffy coat used for DNA extraction via Salting Out technique (according to Miller 1988); however, prior to centrifugation 300 μL aliquots of whole blood were taken for DNA extraction by UltraClean® commercial kit (Mo Bio, USA).

### Obtaining genomic DNA

Isolation of genomic DNA from 17 patients and 30 controls (healthy individuals) was performed by using the Ultra Clean® commercial kit (MO. BIO, USA) and the Salting Out technique (as mentioned). Both extraction methods were used to obtain as much DNA as possible. Amplified DNA products were visualized on 1.5% agarose gels, stained using GelRed ™ (Biotium, USA).

### Polymerase chain reaction (PCR)

Once the genomic material was obtained, sequences comprising the − 1082 (G/A), − 819 (C/T), − 592 (C/A) regions of the human IL-10 gene promoter, the − 590 (C/T) of the IL-4 gene promoter, the Q576R, and Ile50Val substitutions of IL-4Rα, all were amplified via PCR; conditions were adjusted based on those previously described [15, 33] with a final volume of 50 μL.

Amplification products were visualized on 2 and 3% agarose gels stained with GelRed™ (Biotium, USA).

### Genotyping through PCR-RFLP

Amplification products of each segment were subjected to Restriction Fragment Length Polymorphism (RFLP) technique by digestion during 1 h with restriction enzymes for each of the SNPs, as described [15, 33–37]. Digestion products were visualized on 2% agarose gels for all variants, except Q576R, which required a 3% agarose gel [37] and stained with GelRed ™ (Biotium, USA), taking into account the genotypes detailed in Table 1.

**Table 1:** SNP analysis of human cytokines and IL-4Rα

SNP^a^	Cytokine (Promoter/coding sequence)	Restriction enzyme	Genotypes (band size)		
			Wild homozygous	Heterozygous	Homozygous for mutation
- 592 (A/C)	IL-10	RSA I	175 and 237 bp^b^	412, 175 and 237 bp	412 bp
- 819 (T/C)	IL-10	Mae III	209 bp	209, 125 and 84 bp	125 and 84 bp
- 1082(G/A)	IL-10	Mnl I	139 bp	139, 106 and 33 bp	106 and 33 bp
.- 590 (C/T)	IL-4	BsmFI (FaqI)	192 and 60 bp	252, 192 and 60 bp	252 bp
Ile50Val	IL-4Rα	RsaI	273 bp	273, 254 and 19 bp	254 and 19 bp
Q576R	IL-4Rα	MspI	107 and 16 bp	107, 89, 18 and 16 bp	89, 18 and 16 bp

^a^Single-Nucleotide Polymorphism; ^b^ Base pairs

### Control of DNA isolation

The beta chain of human globin was determined in both population groups, patients and controls, to verify the extraction and integrity of the DNA, employing PCR. Forward primer: 5 ‘GGG CAG GTT GGT ATC AAG G 3’ and reverse: 5 ‘AGC CAG GCC ATC ACT AAA 3’ were used, generating a band of 175 bp.

### Determination of cytokine profile: Interleukin 10 (IL-10), gamma interferon (IFN-γ), tumor necrosis factor alpha (TNF-α) and interleukin 4 (IL-4)

One assembly was performed for each cytokine mentioned by employing a sandwich ELISA kit (Affymetrix eBioscience, ThermoFisher Scientific), following manufacturer’sing instructions; whereby 96-well plates were sensitized with each monoclonal antibody capture (overnight at 4 °C followed by three washes); blockage for 1 h with ELISA/ELISPOT buffer 1X (25 °C and three washes) was done; subsequently, each of the eight standard concentration points and unknown samples were incubated for 2 h at 25 °C (followed by three washes per aspiration). A second incubation was carried out using a monoclonal antibody-biotin conjugate (1 h at 25 °C and, subsequently, five washes by aspiration) for detection; then, a third incubation with HRP-Avidin conjugate (30 min at 25 °C and 7 washes per aspiration) was performed; finally, a chromogenic substrate solution was supplied for 15 min (25 °C) and the reaction was stopped by adding H_2_SO_4_ 2 N solution. The microplates were read at 450 nm in a spectrophotometer.

All assemblies were carried out at least in duplicate.

### Statistics

The following were determined as variables: single-nucleotide polymorphism in cytokine gene promoter regions, defined as presence or lack of point change in nitrogenous bases (heterozygote in a single allele or homozygous in two alleles), reflected in the susceptibility to cutting by restriction enzyme, the stage of the disease, understood as its level of progress (qualitative variables), the survival post diagnosis, understood as the time (months) after the diagnosis of gastric carcinoma, and the level of soluble cytokines in peripheral blood, defined as the detectable concentration of protein in pg/mL (quantitative variables).

For the quantitative variables, the average descriptive, standard deviation, median, and range measures were calculated. The age variable was categorized to compare the results, defining two groups: 50 years of age or less, and over 50 years of age bearing in mind that it is a mean age in average adulthood from which changes can be detected in the immune system.

Likewise, the results observed for polymorphisms were classified into two groups: “Mutated” and “Wild”. For the qualitative variables, the calculation of proportions was made by categories of each variable.

The odds ratio (OR) value, the range, and confidence index for the SNP variants, tumour stage and risk of mortality were determined through logistic regression analysis. The comparison was made by estimating the OR without adjusting and 95% CI, as well as the OR adjusted by age and sex variables.

Cytokine serum level (IL-10, TNF-α, INF-γ, and IL-4) was analyzed via Mann-Whitney U-test for comparisons between groups (significant *P*-value ≤0.05 or very significant *P*-value ≤0.01) employing SPSS v19.0 software; the Graphpad Prism v5.0 software was used for graphics. The value of the median, standard deviation, range, and *P-*value was calculated for each variable in each comparison group to determine statistically significant difference.

## Results

Seventeen potentially eligible patients with gastric carcinoma were enrolled in the study, of which 1, 2, and 5 did not complete their molecular analysis for − 592(A/C) SNP of IL-10, − 590 (C/T) SNP of IL-4, and − 1082 (G/A) SNP of IL-10, respectively, due to absence of amplification band after repeated analyses, which is why for the last case paired analysis 1:1 was established with the group of healthy individuals.

Of 31 potentially eligible healthy individuals, 30 were enrolled in the study (one participant excluded due to having samples with signs of deterioration). The description of the population is shown in Table 2.

**Table 2:** Descriptive data of the population

Variable			Patients’ Group^a^ n (%)	Healthy donors’ Group (%) (*n* = 30)
Sex	Female		5 (29.4)	7 (23.4)
	Male		12 (70.6)	23 (76.6)
Age (years)	Mean		61.8	63.5
	Range		(34–90)	(33–82)
Tumour stage^b^ (Borrmann classification)	I		0	Does not apply
	II		5 (38.4)	
	III		4 (30.8)	
	IV		4 (30.8)	
Tumour type^b^ (Lauren classification)	Intestinal-type	Poorly differentiated	3 (23.0)	
		Moderately differentiated	3 (23.0)	
		Well differentiated	3 (23.0)	
	Diffuse		1 (8.0)	
	Not specified		3 (23.0)	
Metastasis^b^	None		7 (53.8)	
	Lymphoid nodules (only)		0	
	Other organs		5 (38.5)	
	Both		1 (7.7)	
Clinical evolution^b^ (Four years later)	Remission (alive)		7 (53.8)	
	Deceased		6 (46.2)	
Survival Post diagnosis^b,g^ Average (range, months)	IL-4 low level^b,c^	Overall	6 (46.2); 33.2 (1–47)	
		Deceased (only)	2 (33.3)^d^; 13.5 (1–26)	
	IL-4 high level^b,e^	Overall	7 (53.8); 26.4 (10–48)	
		Deceased (only)	4 (57.1)^f^; 13.0 (10–18)	
	Absence of IL-10 -1082 (G/A) SNP^g^	Overall	4 (40); 38.8 (26–42)	
		Deceased (only)	1 (25)^h^; 26.0	
	Presence of IL-10 -1082 (G/A) SNP Homozygous or Heterozygous^g^	Overall	6 (60); 21.7 (1–47)	
		Deceased (only)	4 (66.7)^i^; 10.3 (1–18)	

^a^Cases with data, *n* = 17; ^b^ Cases with data, *n* = 13. Data on patients not provided are subject to Institutional policies in keeping with National Legislation on *Habeas data*); ^c^ Less than 5 pg/mL; ^d^ Regarding the totality of patients with low level of IL-4; ^e^ Greater than 5 pg/mL (all cases above 12 pg/mL); ^f^ Regarding the totality of patients with high level of IL-4; ^g^ *n* = 10 (three cases showed no amplification band); ^h^ Regarding the totality of patients with Absence of IL-10 -1082 (G/A) SNP; ^i^ Regarding the totality of patients with Presence of IL-10 -1082 (G/A) SNP

### The − 1082 (G/a) SNP was found associated to gastric carcinoma risk

The mean age in the cases was 61.8 ± 12.9 years and 63.5 ± 11.3 years in the control group, with similar behaviour in both (*P* = 0.670). The male/female ratio was 2.4 and 3.2 in patients and controls, respectively, 70.6% of the cases corresponded to the male gender and 29.4% to the female gender. Information on the characterization of the population analyzed is shown in Table 2.

As shown in Tables 3, 80% of the patients and 90% of the healthy donors had some type of mutation for − 590 (C/T) SNP (IL-4 gene promoter). Although the proportion was higher in the healthy individuals, no significant differences were observed regarding this SNP between groups (*P* = 0.195, 95% CI). In general, no evidence existed of mutation for Ile50Val SNP (IL-4Rα). In relation to the Q576R variant, the mutation rate in the cases was 41.3%, while in the controls it was 43.3%, slightly higher; nevertheless, without significant differences (*P* = 0.600, 95% CI).

**Table 3:** Association measures according to variables of interest

Variable	Cases	Controls	OR^a^ (95% CI^b^) not adjusted	OR (95% CI) ajusted	*P-*value
IL-4 -590 (C/T) SNP (%)	*n* = 15	*n* = 20			
Mutated^c^	12 (80.0)	18 (90.0)	0.44 (0.06–3.07)	0.21 (0.02–2.20)	0.195
Wild	3 (20.0)	2 (10.0)			
IL-4 Ile50Val SNP (%)	*n* = 17	*n* = 30			
Mutated	(0.00)	(0.00)	dna^d^	dna	1.000
Wild	17 (100.0)	30(100.0)			
IL-4 Q576R SNP (%)	*n* = 17	*n* = 30			
Mutated	7 (41.2)	13 (43.3)	0.91 (0.17–3.01)	1.40 (0.37–5.57)	0.601
Wild	10 (58.8)	17 (56.7)			
IL-10 -592(A/C) SNP (%)	*n* = 16	*n* = 29			
Homozygous for mutation	8 (50.0)	14 (48.3)	1.00 (0.32–3.63)	1.00 (0.29–3.77)	0.949
Heterozygous	8 (50.0)	15 (51.7)			
IL-10 -819(T/C) SNP (%)	*n* = 17	*n* = 30			
Mutated	14 (93.3)	27 (90.0)	1.55 (0.15–16.37)	1.50 (0.13–17.18)	0.740
Wild	1 (6.7)	3 (10.0)			
IL-10 -1082 (G/A) SNP (%)	*n* = 12	*n* = 12			
Mutated	6 (50.0)	3 (25.0)	3.00 (0.56–16.89)	7.58 (0.77–74.06)	0.080
Wild	6 (50.0)	9 (75.0)			
IL-4/IL-4Rα SNPs (%)	*n* = 17	*n* = 30			
(≥ 2 mutations)	5 (29.4)	7 (23.3)	1.37 (0.36–5.24)	1.35 (0.34–5.37)	0.673
(< 2 mutations)	12 (70.6)	23 (76.7)			
IL-10 Haplotype (%)	*n* = 16	*n* = 30			
(≥ 2 mutations)	14 (87.5)	26 (86.7)	1.08 (0.17–6.63)	1.08 (0.15–7.42)	0.940
(< 2 mutations)	2 (12.5)	4 (13.3)			

^a^Odds ratio; ^b^ Confidence interval; ^c^ Mutated (heterozygous, homozygous) ^d^ Does not apply

Additionally, for the − 592 (A/C) SNP of the IL-10 gene promoter region, 100% of the cases and controls, which could be measured, had some type of mutation; 50% of the patients had the mutated homozygous genotype and the other 50% had the mutated heterozygous genotype; 48.3% of healthy individuals had the mutated homozygous genotype and 51.7% had the heterozygous genotype. In this sense, although all cases and controls showed a mutation in this gene, no differences were observed (*P* = 0.950, 95% CI).

Regarding the − 819 (T/C) SNP, 6.7% of the patients did not show alterations in this gene but 93.3% had some type of mutation. In the control group, 10% of individuals had no changes; however, 90% had some type of mutation. Likewise, no significant differences were noted between groups (*P* = 0.740, 95% CI).

For the − 1082 (G/A) SNP, the determination could be performed in 14 cases and 22 controls (Some samples from patients did not show any band through PCR for the SNP). To highlight, the mutation risk was twice as high in the group of patients (rate of 50%) compared to healthy individuals (25%), resulting in an adjusted OR value of 7.58 (range 0.77–74.06, 95% CI), with a statistical tendency (*P* = 0.080).

The presence of more than one mutation for both genes was evaluated. It was found that 29.4 and 23.3% of cases and controls, respectively, had mutation in two or more regions of the IL-4/IL-4Rα gene, without significant differences (OR = 1.23; *P* = 0.670).

In turn, for the IL-10 gene promoter region, the same analysis showed that 87.6% of the patients had two or more SNPs (haplotype), whereas in the control group this proportion was 86.7%, with no significant differences (OR = 1.08; *P* = 0.940).

### Patients with gastric carcinoma have increased IL-4 level

For the group of 17 patients, three data items corresponding to the analysis of IL-4 and TNF-α for one case and IL-10 for another were excluded because these exceeded the analytic linearity of the technique; another three cases registered exhaustion of the specimen to quantify IL.10 in serum (one of them additionally for IFN-γ).

With respect to the group of healthy individuals, data from one participant was lost, corresponding to the immunological analysis by sample of icteric serum.

Quantification of circulating IL-4, TNF-α, IL-10 and INF-γ level in 17 patients diagnosed with gastric carcinoma and 30 subjects as control population was achieved by ELISA assay (Affymetrix Ebioscience, USA) using a calibration curve with known concentration standard (as described).

**Fig. 1:**
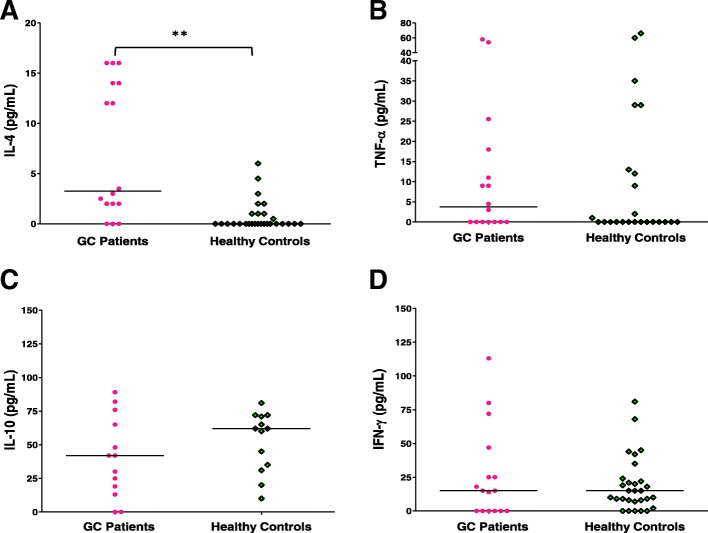
Level of peripheral blood circulating cytokines. The amount of cytokines (pg/mL) is shown: **a** IL-4; (**b**). TNF-α; (**c**). IL-10, and (**b**). IFN-γ in serum from patients with gastric carcinoma, GC (pink filled circles represent each individual) and in healthy population (green empty diamonds per individual). The line corresponds to the median value. *(*P* < 0.05) Statistical significance; **(*P* < 0.01), a very significant value, using the Mann-Whitney U test

Analysis of IL-4 serum level revealed a very significant increase (*P* = 0.001) in its parameter in the patient group, with a median of 3.25 pg/mL (range = 0–16 pg/mL) while it was of 0.00 pg/mL (range = 0–6 pg/mL) in control group (see Fig. 1a). The measurement of TNF-α showed a median of 3.75 pg/mL in patients respect to the control population analyzed in which a median of 0.00 pg/mL (range = 0–66 pg/mL) was observed, with no statistical difference (*P* = 0.530) (see Fig. 1b).

The determination of IL-10 level was performed in 13 patients with gastric carcinoma. The Fig. 1c shows a median of 42 pg/mL (range = 0–89 pg/mL) vs 62 pg/mL (range = 10–81 pg/mL) for patients and control groups, respectively (paired analysis *n* = 13 and 14) with no significant difference (*P* = 0.210).

Finally, when we determine the INF-γ concentration, found the same median of 15 pg/mL in both populations even though different ranges (0–113 pg/mL vs 0–81 pg/mL in cases and controls, respectively), with no statistical difference (*P* = 0.930), as is illustrated in Fig. 1d.

An additional analysis was made taking into account a comparison of circulating cytokines level between patients with recent diagnosis and no treatment yet (*n* = 8 in each subgroup) with regard to those who had already started treatment at the start of the study (to eliminate possible biases due to the effect of antitumor treatment). Data showed in the Table 4.

**Table 4:** Circulating cytokines in subgroups of patients with Gastric Cancer-New Diagnosis (Untreated versus Treated).

	IL-4 (pg/mL)		TNF-α (pg/mL)		IL-10 (pg/mL)		IFN-γ (pg/mL)	
	UT^a^	T^b^	UT	T	UT	T	UT	T
Median	7.25	3.25	3.75	5.50	48.00	27.50	18.00	0.00
Range	0–16	0–16	0–25	0–58	0–89	13–76	0–113	0–72
*P*-value (between subgroups of patients)	0.915		0.661		0.431		0.157	
*P*-value (UT patients versus controls)	**0.001**		0.795		0.377		0.281	

^a^Untreated case; ^b^ Treated. Case

Interestingly, the level of IL-4 (median) in the subgroup of patients recent diagnosed (new or *novo*) and without any treatment was 123% higher than that in treated patients preserving a very significant difference with respect to the serum levels found in healthy individuals.

### Connection between IL-4 level and IL-10 -1082 (G/a) SNP presence to survival in patients

Clinical evolution data were provided for 13 of the patients, with six cases of decease (46.2%). Although the overall survival was 28.1 months, in those cases with higher levels of IL-4 (> 12 pg/mL, *n* = 7), a fatality of 57.1% (4 cases) with an average survival close to 1 year was recorded (Table 2).

Likewise, 3 patients did not show amplification band for the promoter region − 1082 (A / G) of the human IL-10 gene, without established cause, although the analysis was successful in the majority of the analyzed population and was controlled by amplification of the beta chain of human globin, as described; 6 of 10 cases with a result for this variable, evidenced its presence, predominantly heterozygous (5/6 individuals), of which two thirds died with an average survival < 1 year (see Table 2).

Binary logistic regression analysis was carried out with the purpose of establishing a model against the estimation of the risk of mortality from gastric cancer and the presence of the variables of interest such as high level of IL-4 and presence of − 1082 (G/A) SNP in promoter sequence of the IL-10, among others, with its respective coding, using “dummy” variables (Table 5). The omnibus test gave a significant result, which implies that the variables linked to the model to predict the risk of mortality from the disease (*P* = 0.019).

**Table 5:** Coding of included variables

Variables	Category	Frequency	Coding^a^
Age	≤ 50	2	0
	> 50	11	1
Sex	Female	4	0
	Male	9	1
IL-4 pg/mL (level)	Low	6	0
	High	7	1
IL-10 -1082 (G/A) SNP (Presence)	Absent	7	0
	Present	6	1
Stage	II	5	0
	III y IV	8	1
Metastasıs	No	7	0
	Yes	6	1

^a^ The “enter” method was used to classify the results in “dummy” variables and to codify the results of a variable obtained

Table 6 illustrates the summary of the model with the variables introduced. The estimated risk of mortality from gastric cancer in men with respect to women is 2.1, this is also higher for > 50 vs ≤ 50 years, patients with a presence of mutation (2.3 times more likely) and high level of IL- 4 circulating, as well as for those cases with an advanced stage of the tumour. However, the multivariate analysis did not show a statistically significant association between the variables and mortality from gastric cancer (*P* > 0.05), a situation that can be explained by the number of patients linked in the analysis.

**Table 6:** Model summary (multivariate analysis)

Varıable	B	E.T.	Wald	gl	Sig.	Exp(B)^a^
Age(1)	40,3	33,215,2	0,000	1	0,999	3,E+ 17
Sex(1)	0,7	24,452,9	0,000	1	1000	2,1
Soluble IL-4 (1)	21,1	22,579,3	0,000	1	0,999	1,E+ 09
IL-10 -1082 (G/A) SNP (1)	0,8	33,900,2	0,000	1	1000	2,3
Stage (1)	61,6	44,398,0	0,000	1	0,999	6,E+ 26
Metastasıs(1)	−21,7	53,922,6	0,000	1	1000	0,0
Constant	−81,7	50,227,5	0,000	1	0,999	0,0

^a^Estimated values for the OR

## Discussion

In Colombia, where incidence of gastric cancer is high, few studies have sought to correlate some environmental and genetic susceptibility factors with the risk of acquiring this neoplasia [38]. The patients’ group in this study had a mean age of 61.8 ± 12.9 years, where 70.6% of the cases corresponded to the male gender, data consistent with that reported at the national level based on Cancer Population Records and statistics from the National Statistics Department (DANE, for the term in Spanish), revealing a male: female ratio of 1.8:1 [3].

As relevant findings, we show that the SNP variants analyzed, located in the promoter regions of the IL-10 gene: -592 (A/C) and − 819 (T/C), as well as for the IL-4: -590 (C/T) and IL-4Rα (Q576R and Ile50Val), did not present differentially in gastric cancer patients with respect to the group of healthy individuals; even for the last two variants mentioned, homozygous genotypes for the mutation were not evident, so that, at least in the population analyzed, no evidence was noted of any relationship between the presence of these polymorphisms and the development of gastric carcinoma.

These data coincide with those reported in other studies, where the expression of the − 592 (A/C) and − 819 (T/C) variants was very similar between Chinese patients with advanced gastric cancer and the healthy population, without influence, even in their prognosis [33], which does not detract from their clinical relevance, given that in other pathologies, such as squamous intraepithelial cervical lesions and lung cancer, they have been positively associated [39, 40] but not so for − 1082 (G/A). The data is also consistent with that reported by Zhang et al. [41] and recently by Liu et al. [42] with respect to evidence of a relationship between IL-4 -590C/T (rs2243250) SNP and gastric cancer, more likely circumscribed to Asian population.

However, given the histological and molecular heterogeneity registered for gastric carcinoma [43], subsequent in-depth analysis is required that permits correlating the existence of these point mutations with different histological types and their reflexion on the cytokine profile, either circulating or detectable in the tumor microenvironment. We must add to the aforementioned the pertinence of evaluating the association among genetic variants and the probability of developing neoplasia according to the diverse ethnic groups [42]. In that sense, a recent study in the Chinese province of Liaoning reveals, for example, association between the presence of mutation for SNP -819 (C/T) consistent with an ATC haplotype (− 1082/− 819/− 592) and greater risk of having gastric cancer (OR = 3.21; *P* = 0.015) [44], to which Wang et al. [45] add the registry of association among the three SNPs in promoter region of the IL-10 gene, evaluated in our study, and gastric cancer, especially in Asian population, in contrast with that determined for our population. It should be highlighted that said meta-analysis did not register studies from the Colombian population.

Likewise, our results contrast with those by Wu et al. [15] who found association of CT (heterozygous) genotype for the − 590 SNP of the IL-4 promoter region with a risk for aggressive (cardiac) and diffuse gastric carcinoma development (OR = 2.44 and 1.64, respectively) under the hypothesis of a strongly pro-inflammatory environment (therefore, with low IL-4 production) in the tumor bed induced by *H. pylori* infection; our research sought to verify the influence of an anti-inflammatory environment modulated by increased IL-4, possibly of genetic origin (without neglecting other possible origins). Nevertheless, another SNP in IL-4 promoter region has been related to a threefold increased expression of this cytokine, as with − 524 (C/T) [46], whose evaluation merits subsequent consideration.

Our current study showed a significant increase in circulating IL-4 levels (*P* = 0.001) in patients, compared to healthy individuals, a difference that remained intact when the comparison was made by taking into account only patients with new diagnosis and without treatment versus healthy population. The IL-4 level detected in our group of patients is close to that reported by Ock et al. [31] (median of 3.2 Vs. 4.3 pg/mL, respectively), instantly determined in patients with recurrent or metastatic gastric carcinoma, although differing in the value of other cytokines; highlighting, for example, that the serum level of IL-10 was higher in our group of patients (median of 42 Vs. 14.5 pg/mL, respectively) with a considerably higher range for this last study, which revealed a positive correlation among its levels and those of IFN-γ, among other angiogenic factors, with bad prognosis and short survival (10.1 months, *P* = 0.026). Although our results did not evidence association for any of these two cytokines, the average survival found in the group of patients evaluated was similarly low (13 and 10.3 months) although for those cases with a higher level of IL-4 and presence of IL-10 -1082 (G/A) SNP, respectively, which died in a period ≤18 months; as a common characteristic, they had metastasis.

Therefore, the findings in this research could confer relevance to determining this immunological marker in individuals at risk of developing neoplasm, like gastric cancer, in support of our initial hypothesis of a possible environment modulated by IL-4 and M2 cells in patients (whose specific evidence was not within the scope of this study), given that the finding of infiltrating or tumour-associated macrophages (TAMs) in the tumor microenvironment has been associated to poor prognosis [17, 18], evidenced in several neoplasms affecting kidneys, ovaries, endometrium, breasts, lungs, skin [47–53], as well as pancreas, esophageal squamous cell carcinoma [54] and aggressive gastric carcinoma, in relation to a greater stage and poor tumor differentiation [55, 56] although its prognostic role may vary according to the type of tumour [57].

Among other cytokines associated with the Th2 profile, IL-4 has been shown to induce polarization of macrophages to M2 type with immunoregulatory, anti-inflammatory and tumor-promoting activity [57, 58], coherent with prior findings evidencing association between a cytokine hypoproducing phenotype, like IL-6 (OR = 4.8; *P* = 0.002), and the existence of malignant brain tumor, added to the significantly higher expression of the − 1082 (AA) phenotype, IL-10 hypoproducer, in the group of patients with benign neoplasia (OR = 8.0; *P* < 0.001) [16]. Shibata et al. [59] had already shown a decrease in the level of Th1 profile cytokines, like IL-12, in a group of patients with gastric carcinoma (25 cases ranging in age from 32 to 82 years) and advanced colorectal, more significant in those with evidence of metastasis and cachexia, along with a progressive increase for this last group in the level of Th2 profile cytokines, like IL-4 and IL-10, agreeing with diminished Th1/Th2 balance (*P* < 0.05).

Furthermore, Kuehnle et al. [60] evaluated the phenotype of predominant cell populations in peripheral blood from a group of 30 patients with advanced gastric cancer, evidencing very significant increase of three subpopulations of suppressor myeloid cells, two of these at the expense of CD14^+^ cells (*P* < 0.01), along with activated and infiltrating Tregs cells (*P* < 0.01) in the tumor, CD103^+^ (*P* < 0.05), compared with healthy individuals, a presence that has been associated to poor prognosis upon favoring tumor evasion against effective immune response [61], contrary to the prevalent finding of memory cytotoxic T cells within the pool of subpopulations of tumor infiltrating lymphocytes (TIL), biomarker postulated as relevant in solid tissue neoplasia’s, including the digestive tract [62, 63], confirmed – in turn – by the poor prognosis attributed to the high neutrophil-lymphocyte ratio (NLR) in patients with advanced gastric cancer and diminished survival to less than 16 months [64].

Considering that one of the known effects of IL-4, characteristic of Th2 cells, is the polarization of M2 macrophages, which reciprocally promote the response of the first through release of CCL17 and CCL22 chemokines [17], increased circulating IL-4 could merely constitute a reflex of what occurs in the tumor microenvironment with respect to the possible infiltration of suppressor profile TAMs, induced by Th2 lymphocytes, as well as by the tumor itself. This has been evidenced in neoplasia’s, like metastatic breast cancer, based on the production of diverse growth factors, chemokines and, recently, on the Annexin 1 protein, besides de polarization capacity of these macrophages to M2 profile, conferring poor prognosis through progression and angiogenesis [65, 66]. Similarly, description has been made of another coadjuvant role of tumor establishment and metastasis by infiltrating myeloid cells (CD11b^+^), given the production of L-phenylalanine oxidase IL-4 induced gene 1 (IL-4I1), inversely correlated with the presence of cytotoxic T CD8^+^ subpopulations and melanoma control [67].

The presence of M2 macrophages in patients with gastric carcinoma has, likewise, been found associated with poor prognosis, advanced stage of disease and metastasis [20, 55].

The complex and versatile interaction between type M2 TAMs and tumor cells could explain an increased production of IL-4, of induced type and not linked to genetic susceptibility, from the tumor microenvironment.

To highlight, from the genetic perspective, the finding that the mutation frequency for the − 1082 (G/A) SNP of the IL-10 promoter region was twice as high in the patients as compared to healthy individuals (OR = 7.58; *P* = 0.08) determines a strong association for this point mutation with respect to the risk of developing gastric cancer, which could be considered a possible marker of interest in our population. This finding is consistent with that reported in meta-analysis based on studies carried out on the Asian population (in which gastric carcinoma is highly incident), with a connection between the SNP and this neoplasia [68, 69] whose link to other solid tumours, such as cervical cancer and nasopharyngeal carcinoma, in contrast has been rejected [70, 71] (in favor of its specificity as a gastric cancer marker).

According to the aforementioned, this biomarker has also proven relevant in our population, which must be confirmed with subsequent studies involving a larger population.

## Conclusions

We consider that although no association was found among diverse SNPs from human IL-10 and IL-4 promoter regions and gastric cancer event, besides the level of cytokines, like IL-10, TNF-α and IFN-γ, being similar in patients and healthy individuals, the present research provides insight into two possible biomarkers of immunological nature (increased level of serum IL-4) and genetic nature (presence of the IL-10, −G1082A SNP) related to this neoplasm in our population. Of note, the majority (57.1 and 66.7%) of the patients evaluated and with known evolution, which evidenced the first and/or the second biomarker in mention, died in an average period of barely 13 and 10.3 months, respectively. The presence of these two variables revealed a higher estimated risk of mortality in the patients with gastric cancer in our study (multivariate analysis), although without statistical significance.

Our findings contribute to joint efforts in the search for specific and predictive risk markers in the future, given that this disease has been shown to negatively affect the quality of life of patients and the cost of care provided by the health system added to its strong impact upon global [43] and national public health, given that Colombia has much higher rates of incidence and mortality for gastric cancer than other Latin American countries, like Brazil and Mexico [3, 5], which have significantly greater population density and, furthermore, its detection occurs predominantly in advanced stage (with general survival close to one year), whether as a poorly differentiated tumor or with other aggravating factors, like poor therapeutic response due to this neoplasia’s morphological heterogeneity [43, 72]. Although the pathogenesis of gastric cancer is not completely known, factors like genetic susceptibility and immune response turn out relevant to determine its occurrence or evolution [42, 61, 64].

## Abbreviations

CEDMI: Centro Especializado de Diagnٕóstico Materno-Infantil (Acronym in Spanish, for Maternal-Infant Center for Specialized Diagnosis)
CI: Confidence Interval
DNA: Deoxyribonucleic acid
E.S.E: Empresa Social del Estado (Acronym in Spanish, for State Social Enterprise)
EDTA: *Ethylenediaminetetraacetic acid*
ELISA: Enzyme-Linked ImmunoSorbent Assay
HRP: Horseradish peroxidase
HUEM: Hospital Universitario Erasmo Meoz (Acronym in Spanish, for Erasmo Meoz University Hospital)
I.P.S: Institución Prestadora de Salud (Acronym in Spanish for Healthcare Provider Institution)
IFN-γ: Gamma interferon
IL-10: Interleukin 10
IL-4: Interleukin 4
IL-4Rα: Alpha chain of the interleukin-4 receptor
NHL-B: Non-Hodgkin Lymphoma of B cells
OR: Odds ratio
PCR-RFLP: Polymerase chain reaction- restriction fragment length polymorphism
SNP: Single-Nucleotide Polymorphism
TAM: Tumor-associated macrophages
Th1: T helper 1 cell
Th2: T helper 2 cell
TNF-α: Tumor necrosis factor alpha
UDES: Universidad de Santander
WMA: World Medical Association
